# Structure of the SPRY domain of the human RNA helicase DDX1, a putative interaction platform within a DEAD-box protein

**DOI:** 10.1107/S2053230X15013709

**Published:** 2015-08-25

**Authors:** Julian N. Kellner, Anton Meinhart

**Affiliations:** a Department of Biomolecular Mechanisms, Max Planck Institute for Medical Research, Jahnstrasse 29, 69120 Heidelberg, Germany

**Keywords:** DEAD-box proteins, SPRY domains, RNA processing, protein–protein interaction

## Abstract

The human RNA helicase DDX1 in the DEAD-box family plays an important role in RNA processing and has been associated with HIV-1 replication and tumour progression. Whereas previously described DEAD-box proteins have a structurally conserved core, DDX1 shows a unique structural feature: a large SPRY-domain insertion in its RecA-like consensus fold. SPRY domains are known to function as protein–protein interaction platforms. Here, the crystal structure of the SPRY domain of human DDX1 (hDSPRY) is reported at 2.0 Å resolution. The structure reveals two layers of concave, antiparallel β-sheets that stack onto each other and a third β-sheet beneath the β-sandwich. A comparison with SPRY-domain structures from other eukaryotic proteins showed that the general β-sandwich fold is conserved; however, differences were detected in the loop regions, which were identified in other SPRY domains to be essential for interaction with cognate partners. In contrast, in hDSPRY these loop regions are not strictly conserved across species. Interestingly, though, a conserved patch of positive surface charge is found that may replace the connecting loops as a protein–protein interaction surface. The data presented here comprise the first structural information on DDX1 and provide insights into the unique domain architecture of this DEAD-box protein. By providing the structure of a putative interaction domain of DDX1, this work will serve as a basis for further studies of the interaction network within the hetero-oligomeric complexes of DDX1 and of its recruitment to the HIV-1 Rev protein as a viral replication factor.

## Abstract 2

The structure of the SPRY domain of the human RNA helicase DDX1 was determined at 2.0 Å resolution. The SPRY domain provides a putative protein–protein interaction platform within DDX1 that differs from other SPRY domains in its structure and conserved regions.

## 1. Introduction   

The human RNA helicase DDX1 (DEAD-box protein 1) was originally identified by its overexpression in neuroblastoma and retinoblastoma cell lines and is a ubiquitous player in RNA processing that is prevalent in all cell types (Godbout & Squire, 1993 ▸; Godbout *et al.*, 1998 ▸). DDX1 belongs to the subgroup of DEAD-box proteins within the superfamily 2 (SF2) of nucleic acid helicases, characterized by the conserved DEAD sequence motif (Linder *et al.*, 1989 ▸). DDX1 differs from all other DEAD-box proteins in a novel structural feature, a SPRY insertion domain in the helicase core (Godbout *et al.*, 1994 ▸). The core fold of DEAD-box proteins consists of two similar and tandemly arranged RecA-like globular α/β-domains that harbour up to 14 conserved motifs (Jankowsky & Fairman, 2007 ▸). In DDX1, this DEAD-box core fold is disrupted by the large SPRY domain, prominently positioned in RecA-like domain 1 between the P-loop (Walker A) motif and conserved motif Ia (Supplementary Fig. S1; Godbout *et al.*, 1994 ▸), which separates the motifs by around 240 residues instead of the usual to 20–40 residues as in other DEAD-box proteins (Schmid & Linder, 1992 ▸). The only other DEAD-box protein with an insertion at the same position is human DDX3X (PDB entry 2i4i; Högbom *et al.*, 2007 ▸), but here the insertion consists of only ten residues. DDX1 has been found to be a component of the hetero-pentameric HSPC117 complex (Trowitzsch, 2008 ▸) that functions in tRNA processing (Popow *et al.*, 2011 ▸, 2014 ▸) and RNA transport (Kanai *et al.*, 2004 ▸; Pérez-González *et al.*, 2014 ▸). Moreover, DDX1 is associated with the formation and progression of germ-cell tumours (Godbout *et al.*, 2007 ▸; Tanaka *et al.*, 2009 ▸) and has been suggested to be useful as a potential biomarker in breast cancer (Balko & Arteaga, 2011 ▸). Furthermore, DDX1 is hijacked as a host factor in several viral replication pathways, the most prominent being HIV-1 mRNA trafficking *via* the Rev–CRM1 pathway (Fang *et al.*, 2004 ▸, 2005 ▸; Yedavalli *et al.*, 2004 ▸). DDX1 binds to the HIV-1 Rev protein and the Rev response-element RNA (Edgcomb *et al.*, 2011 ▸) and promotes the export of unspliced mRNA (Robertson-Anderson *et al.*, 2011 ▸). Although several studies have shed light on these different cellular functions of DDX1, the functional relevance of the SPRY-domain insertion within the protein core remains uncharacterized. SPRY domains are common protein–protein interaction domains that were originally identified as a sequence repeat in the dual-specificity kinase SplA (SP) and Ca^2+^-release channel ryanodine (RY) receptors (Ponting *et al.*, 1997 ▸). Ryanodine receptors (RyRs) contain three SPRY domains that are conserved from invertebrates to vertebrates (Lau & Van Petegem, 2014 ▸; Perálvarez-Marín *et al.*, 2011 ▸). SPRY domains share a notable sequence homology with the longer B30.2 domains. Many SPRY domains contain an additional β-sheet at the N-terminus consisting of three β-strands, termed the PRY domain, resulting in a similar overall domain architecture to B30.2 domains (Woo, Suh *et al.*, 2006 ▸; D’Cruz, Babon *et al.*, 2013 ▸). Despite only moderate sequence homology of these additional N-terminal extensions to the B30.2 domain, they are structurally very similar to the respective B30.2 N-terminus. This led to the collective designation of the combined PRY/SPRY and B30.2 domains as SPRY domains. SPRY domains are present in more than 100 human proteins that can be subdivided into 11 protein families (Rhodes *et al.*, 2005 ▸). Some structures of SPRY domains from the SOCS-box family of E3 ubiquitin ligases (SPSBs; Filippakopoulos *et al.*, 2010 ▸; Woo, Imm *et al.*, 2006 ▸) and TRIM proteins (Biris *et al.*, 2012 ▸; D’Cruz, Kershaw *et al.*, 2013 ▸; James *et al.*, 2007 ▸; Keeble *et al.*, 2008 ▸; Park *et al.*, 2010 ▸; Yang *et al.*, 2012 ▸) have been reported. In addition, the structure of the *Drosophila* SPSB protein GUSTAVUS in complex with a short interacting peptide led to the identification of a conserved interaction platform, surface A, that is present in many SPRY domains (Woo, Imm *et al.*, 2006 ▸; Woo, Suh *et al.*, 2006 ▸; Styhler *et al.*, 2002 ▸; Filippakopoulos *et al.*; Kuang *et al.*, 2009 ▸). Similarly, an analogous interaction site has been identified in structural studies of SPRY domains of TRIM21 proteins (D’Cruz, Kershaw *et al.*, 2013 ▸; James *et al.*, 2007 ▸). In contrast, a positively charged surface, located at a different site to surface A, was suggested to be the interaction region of the SPRY domain of Ash2L, a regulator of histone methylation (Chen *et al.*, 2012 ▸; Woo, Suh *et al.*, 2006 ▸; James *et al.*, 2007 ▸).

Here, we report the crystal structure of the SPRY domain of human DDX1 (hDSPRY) and provide the first structural information on the DEAD-box protein DDX1. We discuss its potential function as a protein–protein interaction domain and how hDSPRY potentially serves to recruit DDX1 to various protein complexes. DDX1 is ubiquitous in the cell and is involved in viral replication and overexpressed in tumour cells. Thus, understanding its interaction with other proteins could be of therapeutic relevance. The presented structure of hDSPRY will lay the foundation for a profound characterization of the atomic details of the DDX1 interaction network.

## 2. Materials and methods   

### 2.1. Protein cloning, expression and purification   

The coding sequence of human DDX1 was amplified from cDNA (obtained from Open Biosystems; accession No. BC012739, clone ID 3835131) and cloned into pET-28a expression vector (Novagen) as described previously (Kellner *et al.*, 2015 ▸). For cloning of the coding sequence for the hDSPRY domain, a BamHI restriction site was introduced upstream and an XhoI restriction site downstream of the potential domain (encoding amino acids 72–283 of DDX1) following the QuikChange site-directed mutagenesis (SDM) protocol (Agilent Technologies). The mutated plasmid DNA was digested with BamHI and XhoI restriction endonucleases and the excised fragment was ligated into pET-28a vector, resulting in the construct pET-28a(SPRY_72–283+Tag) carrying an N-terminal hexahistidine tag. Several additional variants that were N-terminally truncated [pET-28a(SPRY_84–283+Tag) and pET28a(SPRY_100–283+Tag)], C-terminally truncated [pET28-a(SPRY_72–261+Tag)] or truncated at both termini [pET-28a(SPRY_84–261+Tag) and pET-28a(SPRY_100–261+Tag)] were produced (Supplementary Table S1). Nucleotides in the 5′ region of the SPRY-coding sequence were removed by the introduction of BamHI restriction sites upstream and downstream of the respective nucleotides *via* SDM, subsequent BamHI restriction-endonuclease digestion, removal of the BamHI–BamHI restriction fragment and re­ligation of the vector. Nucleotides in the 3′ region of the SPRY-coding sequence were removed by the introduction of stop codons *via* SDM. All cloning steps were verified by sequencing (MWG Eurofins); the primers are listed in Supplementary Table S2.

The recombinant protein was expressed in *Escherichia coli* BL21-CodonPlus(DE3) RIL cells (Invitrogen) grown in Luria–Bertani medium at 310 K and protein expression was induced at an OD_600_ of 0.6 with 0.5 m*M* isopropyl β-d-1-thiogalacto­pyranoside after cooling the cell culture to 293 K. Cells were grown for 14 h and harvested by centrifugation. The cell pellets were resuspended in lysis buffer (50 m*M* Tris–HCl pH 8.0, 250 m*M* KCl, 10 m*M* β-mercaptoethanol) and lysed by sonication on ice with a Branson sonifier. The lysate was cleared at 125 000*g* at 277 K for 40 min and the supernatant was loaded onto an Ni–NTA column (GE Healthcare) pre-equilibrated with lysis buffer. The recombinant protein was eluted using lysis buffer containing an additional 250 m*M* imidazole. The protein-containing fractions were pooled and either digested with 200 U thrombin overnight at 277 K to remove the N-terminal hexahistidine tag or directly diluted in buffer *A*_hep_ [50 m*M* Tris–HCl pH 8.0, 5 m*M* MgCl_2_, 3 m*M* 1,4-dithioerythritol (DTE)] and loaded onto a 1 ml HiTrap heparin column (GE Healthcare) at 277 K using an ÄKTApurifier 10 (GE Healthcare). The bound protein was eluted with a linear gradient to 1 *M* KCl and the protein-containing fractions were diluted in buffer *A*_monoS_ (50 m*M* MES–NaOH pH 5.5, 5 m*M* MgCl_2_, 3 m*M* DTE) and loaded onto a Mono S 5/50 GL column (GE Healthcare). The protein was eluted with a linear gradient to 1 *M* KCl and protein-containing fractions were concentrated using Amicon Ultra 10K MWCO filters (Millipore). Further purification was achieved by size-exclusion chromatography on a Superdex S75 10/300 GL column (GE Healthcare) equilibrated in storage buffer (10 m*M* HEPES–NaOH pH 8.0, 250 m*M* KCl, 5 m*M* MgCl_2_, 3 m*M* DTE). Fractions containing pure SPRY protein were pooled, concentrated using Amicon Ultra 10K MWCO filters and stored in 20 mg ml^−1^ (780 µ*M*) aliquots at 193 K for crystallization. Protein concentrations were determined spectroscopically using the absorbance at 280 nm (∊ = 25 440 *M* ^−1^ cm^−1^ for the SPRY_72–283 construct). All purification steps were verified by 15%(*w*/*v*) SDS–PAGE with Coomassie Blue staining. Purified SPRY protein was confirmed to run as a single band with a molecular weight of 28 kDa and its identity was confirmed by MALDI-MS. Selenomethionine-substituted protein was expressed according to Van Duyne *et al.* (1993 ▸) and protein purification was performed essentially as described for the native protein, except that the concentration of DTE in the *A*_hep_, *A*_monoS_ and storage buffers was increased to 5 m*M*.

### 2.2. Protein characterization by CD melting and dynamic light scattering   

**Figure 1:**
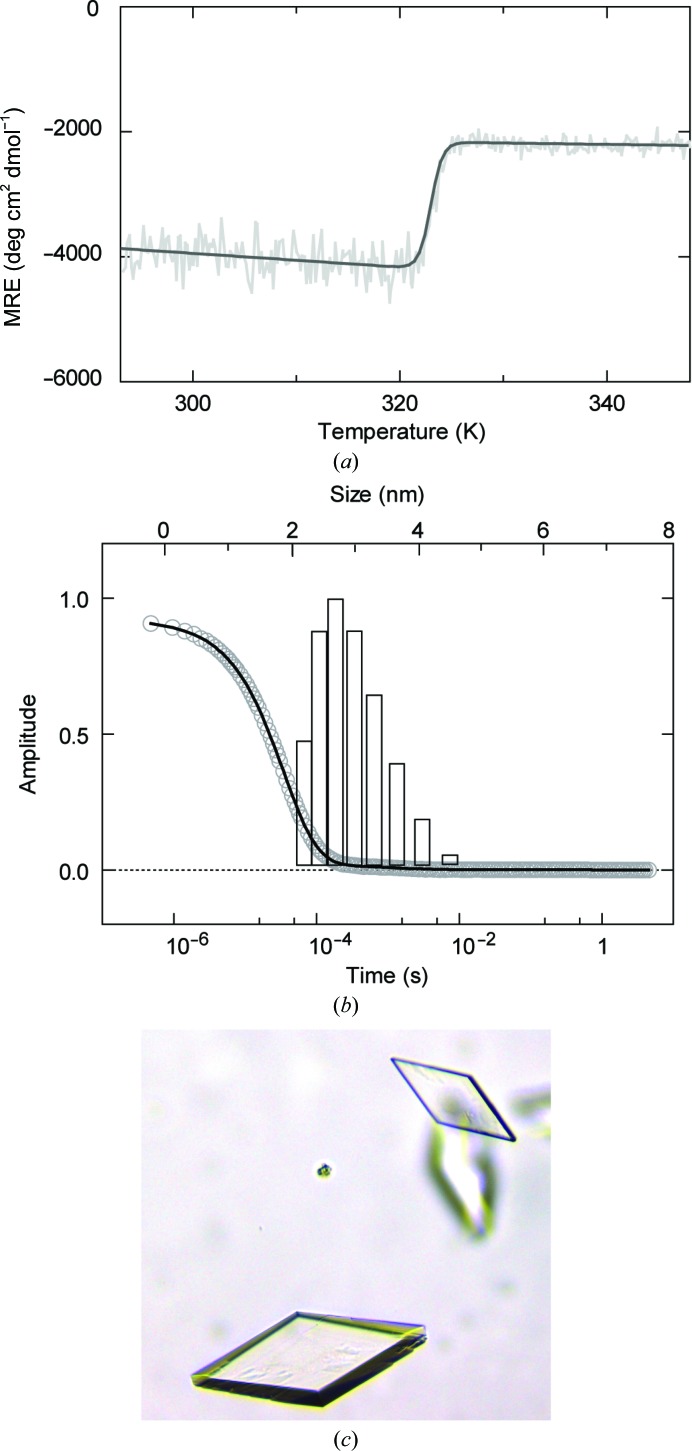
Biophysical characterization and crystals of the SPRY_72–283 construct. (*a*) Thermal denaturation of SPRY_72–283 monitored by recording the CD signal at 222 nm. Data are given as the mean residue ellipticity (MRE) and were fitted to a two-state unfolding process, yielding an apparent *T*_m_ of 323 K. Note that the CD signal is intrinsically low owing to the pure β-strand content of hDSPRY. (*b*) DLS measurements of SPRY_72–283. The lower *x* axis refers to the combined autocorrelation function (grey circles), with the corresponding fit shown as a black line. The upper *x* axis refers to the distribution of the hydrodynamic radii by relative mass (the amplitude of each bar indicates the share of the total mass of the sample) as obtained from the fit. The graph shows a peak at a hydrodynamic radius of 2.8 nm with a peak width of 18% relative standard deviation, which indicates a high degree of sample homogeneity. (*c*) Crystal of the SPRY_72–283 protein construct used to determine the structure of hDSPRY.

The stability of the SPRY domain was characterized by thermal denaturation monitored by a Jasco J-810 circular-dichroism (CD) spectropolarimeter. A solution of 130 µg ml^−1^ (5 µ*M*) protein in CD buffer (50 m*M* K_2_HPO_4_/KH_2_PO_4_ pH 8.0, 250 m*M* KF, 3 m*M* DTE) was heated from 293 to 368 K at a rate of 1 K min^−1^ and unfolding was followed by recording the light polarization at 222 nm. The buffer and wavelength were chosen to optimize the CD signal from the β-sheet structure (Supplementary Fig. S2). Melting curves were fitted to a two-state unfolding equation (Santoro & Bolen, 1988 ▸; Fig. 1 ▸ *a*).

The homogeneity and oligomeric state of the protein were characterized *via* dynamic light scattering (DLS). 650 µg ml^−1^ (25 µ*M*) protein in storage buffer was measured in a Viscotek 802 (Malvern Instruments), which records scattered light at a 90° angle. 30 light-fluctuation curves with 4 s measurement time each were recorded. All traces with constant intensity were averaged to fit a combined auto-correlation function, from which the hydrodynamic radius was extracted (Fig. 1 ▸ *b*). All DLS data analysis was performed using the *OmniSIZE* software (Malvern Instruments).

### 2.3. Domain mapping *via* limited proteolysis   

Limited proteolysis experiments were performed by digesting protein samples with commercially available proteases (Sigma–Aldrich). A sample of 1.82 mg ml^−1^ (70 µ*M*) protein in storage buffer was supplemented with 4 m*M* CaCl_2_ and 2.5 µg trypsin, chymotrypsin or thermolysin. Reactions were incubated at 310 K and quenched at defined time points by freezing aliquots in liquid nitrogen; digestion products were separated by 15%(*w*/*v*) SDS–PAGE. Protein bands were excised from the gel and analyzed by MALDI-MS.

### 2.4. Construct design, screening and crystallization   

Sequence alignment of the human DDX1 with other DEAD-box proteins was used to determine the domain boundaries of the SPRY domain (amino acids 72–283 of DDX1; see Supplementary Fig. S3). This region was further analyzed by bioinformatic tools to guide and refine the design of stable constructs suitable for structural studies. Secondary-structure prediction (*PSIPRED*; Buchan *et al.*, 2010 ▸) showed that the N- and C-terminal residues are potentially flexible and might not adopt a defined secondary structure. Sequence alignment of DDX1 orthologues (Supplementary Fig. S4) showed that the residues located N- and C-terminal to the SPRY domain are not conserved and further suggested that these residues may constitute linker regions that connect the SPRY domain to the RecA-like domain 1 of the DEAD-box core. In order to facilitate protein crystallization, several constructs with varying lengths of the N- and C-termini were designed. All hDSPRY constructs are summarized in Supplementary Table S1. Domain boundaries were based on limited proteolysis experiments and constructs were tested for expression and protein solubility. Constructs that were stable and homogeneous in solution were used for screening.

Crystallization experiments were performed with soluble constructs by sitting-drop vapour diffusion using commercially available crystallization screens (Qiagen) at 293 K. Experiments were set up in 96-well XTL low-profile plates (Greiner Bio-One) by mixing 100 nl protein sample at 20 mg ml^−1^ (780 µ*M*) with 100 nl reservoir solution using a Mosquito robotic system (TTP Labtech). Crystals of the SPRY_84–261+Tag construct were obtained in various conditions using the JCSG Core Suites (Qiagen). The largest crystals grew in 30%(*w*/*v*) PEG 3000, 0.1 *M* CHES–NaOH pH 9.5. Crystals of the same construct but with selenomethionine incorporated were obtained under the same conditions. The protein construct SPRY_72–283+Tag, which presumably had the longest unstructured termini attached to the SPRY core, was also stable and could be purified to homogeneity (Figs. 1 ▸ *a* and 1 ▸ *b*). This variant also crystallized after 3 d in a reservoir solution consisting of 40%(*v*/*v*) PEG 600, 0.1 *M* trisodium citrate pH 5.5. This condition was refined by hanging-drop vapour diffusion using 24-well Linbro plates (Greiner Bio-One), mixing 1 µl protein solution with 1 µl reservoir solution. Small crystals which were obtained after 3 d were crushed and used for streak-seeding into fresh protein/reservoir setups. We could further optimize the crystal size by removal of the N-terminal hexahistidine tag (SPRY_72–283). After final optimization, the SPRY_72–283 construct showed crystal formation in a reservoir solution consisting of 35%(*v*/*v*) PEG 600, 0.1 *M* trisodium citrate pH 5.5. Spheroid crystals appeared after 3 d and grew as single crystals with typical dimensions of 140 × 90 × 40 µm within 6 d (Fig. 1 ▸ *c*). For data collection, single crystals of the SPRY constructs were soaked in reservoir solution for cryoprotection. For crystals of the SPRY_84–261+Tag construct, an additional 20%(*v*/*v*) glycerol was required for sufficient cryoprotection. Subsequently, crystals were harvested and flash-cooled in liquid nitrogen.

### 2.5. Crystal screening, data collection, structure determination and refinement   

Diffraction data were collected on a Pilatus 6M detector (Dectris) on beamline X10SA at the Paul Scherrer Institute (PSI), Villigen, Switzerland. The largest crystals that were produced from the SPRY_84-261+Tag construct diffracted to a resolution of 2.7 Å and belonged to space group *P*1. The Matthews coefficient (Matthews, 1968 ▸) of 2.34 Å^3^ Da^−1^ suggested that ten molecules were located in the asymmetric unit. In addition, the self-rotation function calculated with *MOLREP* (Vagin & Teplyakov, 2010 ▸) from the *CCP*4 suite (Collaborative Computational Project, Number 4, 1994 ▸; Winn *et al.*, 2011 ▸) did not reveal any strong noncrystallographic rotational symmetry and the native Patterson calculated with *FFT* (Ten Eyck, 1973 ▸) did not show any sign of translational symmetry. It is most likely that the combination of these unfavourable conditions caused attempts to phase the data set by molecular replacement to fail. On the other hand, the small crystals of the SPRY_72–283+Tag construct did diffract but only to approximately 4.0 Å resolution. However, removal of the N-terminal hexahistidine tag (SPRY_72–283) significantly improved the diffraction quality, and diffraction data from protein crystals of this construct were used to determine the molecular structure to a resolution of 2.0 Å. All screening results from the different SPRY constructs are summarized in Supplementary Table S1.

The data were indexed, integrated and scaled using *XDS* (Kabsch, 1993 ▸; Table 1 ▸). A rather strict resolution cutoff was applied at 2 Å for the crystals of the SPRY_72–283 constructs, as the reflections in the next highest resolution shell (2.0–1.8 Å) revealed an *R*_meas_ higher than 100%, although the signal-to-noise ratio was still high. For the calculation of *R*_free_ (Brünger, 1992 ▸), 5% of the reflections were randomly assigned and omitted during refinement. Initial phases were obtained by molecular replacement (MR) with *Phaser* (McCoy *et al.*, 2007 ▸) using diffraction data between 47 and 2.5 Å resolution with the SPRY domain of Ash2L as a search model (Chen *et al.*, 2012 ▸; PDB entry 3toj; 23.8% sequence identity). To reduce the model bias inherently introduced by molecular replacement, parts of the model that could be unambiguously rebuilt into the electron density obtained after *Phaser* molecular replacement were corrected to the sequence of the SPRY_72–283 domain. Regions that were less defined or where the electron density was ambiguous were deleted from the model. This initial model was then used for refinement, and phase extension to 2.0 Å by simulated annealing in *CNS* (Brünger *et al.*, 1998 ▸) was performed. Subsequently, the model was completed and refined by iterative cycles of manual building using *Coot* (Emsley *et al.*, 2010 ▸) followed by simulated annealing. Subsequent stages of refinement were carried out with *REFMAC* (Murshudov *et al.*, 2011 ▸) using *TLS* (Winn *et al.*, 2001 ▸) within the *CCP*4 suite (Winn *et al.*, 2011 ▸; Collaborative Computational Project, Number 4, 1994 ▸) and manual improvement in *Coot*, leading to a final model with an *R* factor of 20.0% and an *R*_free_ of 23.7% (Table 1 ▸, Supplementary Fig. S5). Water molecules were assigned manually by selecting electron-density peaks above 3σ in the *F*_o_ − *F*_c_ difference map with correct hydrogen-bonding distances and coordination. Sodium ions were identified based on an octahedral coordination sphere. The quality of intermediate and final structures was evaluated using *MolProbity* (Chen *et al.*, 2010 ▸) and *PROCHECK* (Laskowski *et al.*, 1993 ▸). All structural representations were generated using *PyMOL* (DeLano, 2002 ▸) with subsequent ray tracing. Electrostatic surface potentials were calculated with the *PyMOL* plug-in *APBS* (Baker *et al.*, 2001 ▸).

## 3. Results and discussion   

### 3.1. Overall structure of the SPRY domain   

We have determined the crystal structure of the complete SPRY domain (amino acids 86–279) of the human DEAD-box protein DDX1 at a resolution of 2.0 Å (Table 1 ▸). The final crystallization construct SPRY_72–283 showed a melting point of 323 K and a DLS mass-distribution peak corresponding to a homogeneous sample with a hydrodynamic radius of 2.8 nm (Figs. 1 ▸ *a* and 1 ▸ *b*). Assuming a spherical shape of the molecule, this would lead to a calculated mass of 41 kDa, which is higher than the calculated monomeric mass of 28 kDa for SPRY_72–283 but is too low to assume dimerization. However, SPRY_72–283 is more likely to be a monomer, as the entire hDDX1 protein has been shown to be monomeric (Kellner *et al.*, 2015 ▸). The construct SPRY_72–283 crystallized within 3 d (Fig. 1 ▸ *c*) and the crystals belonged to space group *P*2_1_2_1_2_1_, with two molecules of hDSPRY per asymmetric unit (chains *A* and *B*) and a solvent content of 49.5%. As indicated by a strong translational peak at (0.00, 0.50, 0.04) in the native Patterson, the two molecules were related by a noncrystallographic twofold rotational symmetry axis that is nearly parallel to the twofold screw axis along *b*. Thus, it was unclear whether the choice of origin in space group *P*2_1_2_1_2_1_ was correct or a whether a pseudo-origin had been chosen. The data were re-indexed in space group *P*2_1_2_1_2 assuming a non­crystallographic twofold screw axis along *c*. Although a solution was found by MR, further refinement did not converge, showing that the presumed *P*2_1_2_1_2 origin represents a pseudo-origin.

The protein construct used in the final crystallization setup consisted of residues 72–283 of human DDX1; however, clear electron density was only observed for residues 86–275 of chain *A* and residues 86–279 of chain *B*. The residues at the N- and C-terminus that could not be modelled are likely to be disordered in the crystal since we observed some ambiguous density that could not be interpreted. The models for the polypeptide chains *A* and *B* have a C^α^ r.m.s. deviation of 0.256 Å for alignment of all 193 residues (an r.m.s. deviation of 1.239 Å for all atoms), indicating that they are almost identical. Since the models of the two molecules of hDSPRY only differ in four residues at the C-terminus that could not be unambiguously modelled in chain *A*, the following discussion and figures will be based on the model of chain *B*.

**Figure 2:**
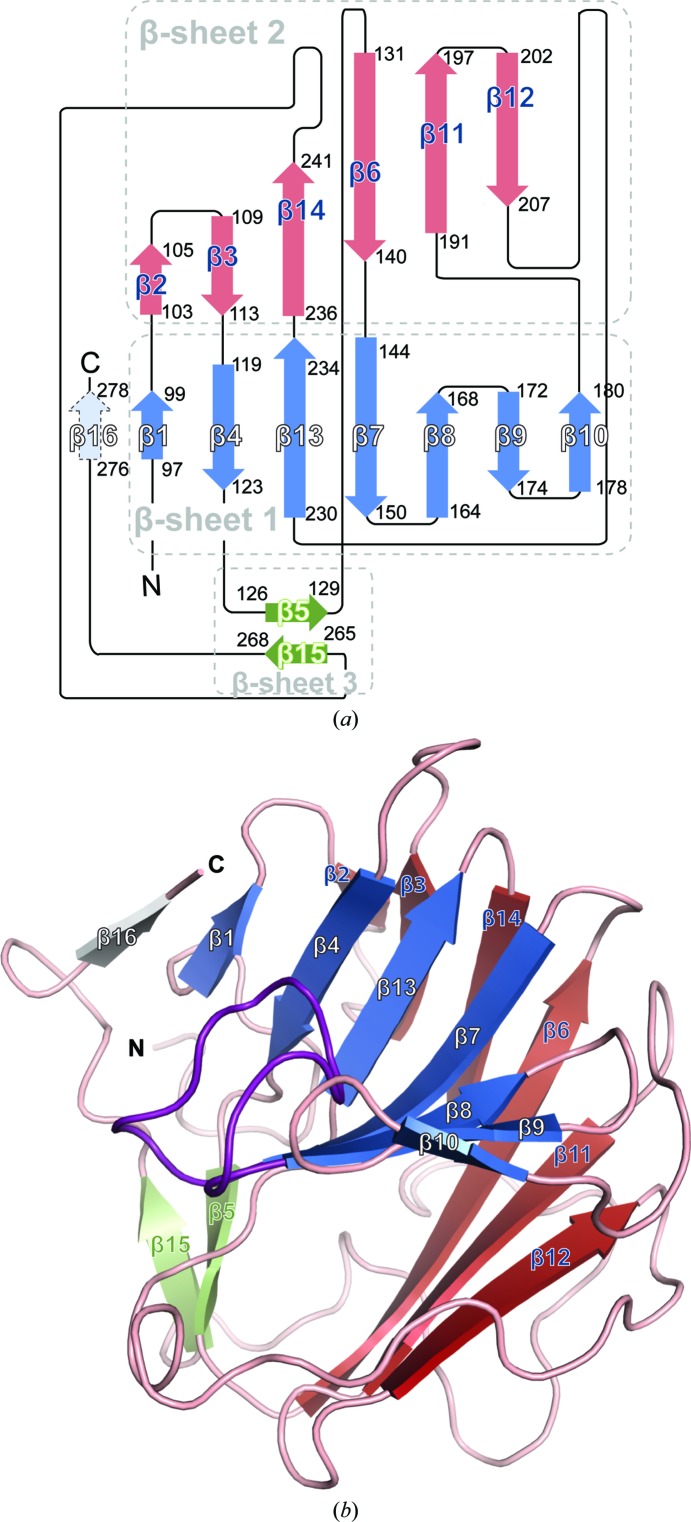
Structure and topology of hDSPRY. (*a*) Topology map with β-sheet 1 coloured blue, β-sheet 2 red and β-sheet 3 green. β-Strands are illustrated as arrows. The artificial β-addition module, β-strand 16, of chain *B* is shown in grey. (*b*) The β-sandwich fold of hDSPRY; colouring is similar to that in (*a*). Loop D is highlighted in purple.

hDSPRY adopts a compact β-sandwich conformation. All secondary-structure elements form β-strands (Fig. 2 ▸ *a*) and in contrast to other SPRY domains (Chen *et al.*, 2012 ▸; Park *et al.*, 2010 ▸; D’Cruz, Kershaw *et al.*, 2013 ▸; Weinert *et al.*, 2009 ▸) no α-helical regions could be found in the N- and C-termini. The β-sandwich fold is slightly twisted and forms a bowl-like platform. Two layers of concave β-sheets stack together and are referred to in the following as β-sheets 1 and 2, and a third small β-sheet covers one side of the β-sandwich (Fig. 2 ▸). β-Sheet 1 is composed of eight strands (β16, β1, β4, β13, β7, β8, β9 and β10), β-sheet 2 is composed of six strands (β2, β3, β14, β6, β11 and β12) and the small β-sheet 3 consists of only two strands (β15 and β5). All β-strands of the β-sandwich core are arranged in an antiparallel configuration, except for strands β16 and β1, which are oriented parallel. Interestingly, strand β16 is only observed in the model of chain *B* as it consists of the four additional residues that could be modelled at the C-terminus (residues 276–279) of chain *A*. It is held in place by hydrogen bonds to strand β1 and forms a β-addition module, which might be an artifact of crystal packing. On one hand, the residues of strand β16 in the model of chain *B* do not belong to the SPRY core domain (Fig. 2 ▸ *a* and Supplementary Fig. S4). On the other hand, the C-terminal loop region in the model of chain *A* adopts a totally different conformation to that in chain *B* and points away from the SPRY domain.

The long loop regions mainly cluster on one side of the β-sandwich, which is adjacent to β-sheet 3, in particular the loop between β12 and β13 (23 residues) as well as that between β14 and β15 (24 residues) (Fig. 2 ▸). The third long loop situated between β-strands β7 and β8 (14 residues; shown in purple in Fig. 2 ▸ *b*) is commonly observed in the structures of other SPRY domains (D’Cruz, Babon *et al.*, 2013 ▸) and has been termed ‘loop D’ (Woo, Suh *et al.*, 2006 ▸). Loop D lies in the bowl-like curvature of β-sheet 1 and covers a hydrophobic patch on this concave side of the β-sandwich. Similarly, on the other, convex, side of the β-sandwich the loop connecting β-strands β14 and β15 shields the hydrophobic patch on β-sheet 2 and has been termed the ‘lid’ in recently published RyR SPRY structures (Lau & Van Petegem, 2014 ▸; Supplementary Fig. S6).

### 3.2. Interface between the two layers of β-sheets   

**Figure 3:**
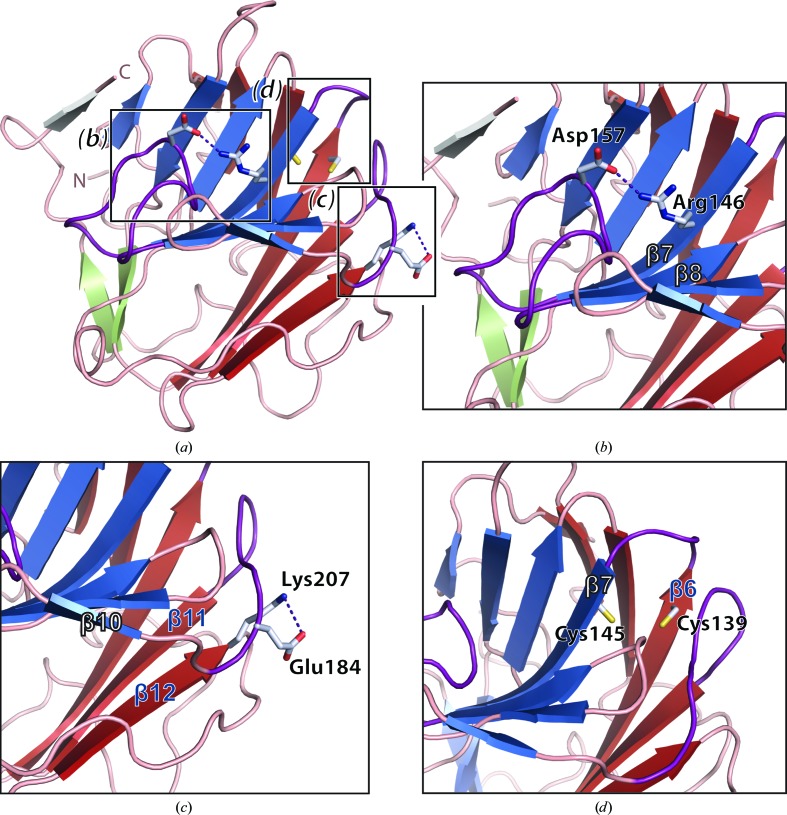
Interactions within the β-sandwich fold. (*a*) Overview of the hDSPRY structure, with the regions displayed in the enlargements in (*b*), (*c*) and (*d*) highlighted. Residues that are discussed in the manuscript are shown as stick models. (*b*) Loop D lies in a bowl-like curvature and forms a salt bridge between Arg146 and Asp157. (*c*) The salt bridge between Glu184 and Lys207 in the loop connecting β10 and β11 is depicted in purple. (*d*) Cys139 and Cys145 that do not form a disulfide bond are shown.

The residues at the interface of the two β-sheets play an important role in maintaining the structural integrity of SPRY domains (Grütter *et al.*, 2006 ▸; Chen *et al.*, 2012 ▸). The strands from β-sheet 1 encompass an intramolecular hydrophobic core with the opposite strands of β-sheet 2. Hydrophobic residues from strands β2, β6, β7, β8, β11, β12, β13 and β14 stack together through van der Waals interaction. In addition to these hydrophobic interactions, salt bridges and hydrogen bonds also contribute to the interaction of the β-sheets and potentially to conformational rigidity (Fig. 3 ▸ *a*). The main-chain amide and carbonyl of Gly148 are at a hydrogen-bonding distance from the O atom of the side chain of Tyr135 (3.5/2.8 Å). The side-chain amide group of Lys173 is at a hydrogen-bonding distance from the main-chain carbonyl group of Ala215 (2.6 Å). A salt bridge is formed between the side-chain guanidine moiety of Arg146 and the side-chain carbonyl group of Asp157 (2.7 Å; Fig. 3 ▸ *b*). Another salt bridge is formed between the side-chain carbonyl group of Glu184 and the side-chain amide group of Lys207 (3.5 Å; Fig. 3 ▸ *c*). Interestingly, the thiol groups of Cys139 and Cys145 adopt conformations such that the distance between the S atoms is only 4.3 Å (Fig. 3 ▸ *d*), but do not form a disulfide bond. Despite this cysteine proximity there is no evidence of any residual electron density for a disulfide bond partially opened by radiation damage (Sutton *et al.*, 2013 ▸).

### 3.3. Structural comparison with other SPRY domains   

To date, only a limited number of SPRY domains from other eukaryotic proteins have been structurally characterized (Filippakopoulos *et al.*, 2010 ▸; Grütter *et al.*, 2006 ▸; Weinert *et al.*, 2009 ▸), and a structural comparison using the *DALI* web server (Holm & Rosenström, 2010 ▸) identified high structural similarity of hDSPRY (residues 72–283) to the SPRY domains of the human trithorax protein Ash2L (PDB entry 3toj; Chen *et al.*, 2012 ▸), mammalian RyR1 and RyR2 (PDB entries 4p9j and 4p9i; Lau & Van Petegem, 2014 ▸), human SPSB proteins 1, 2 and 4 (PDB entries 2jk9, 3emw and 2v24; Filippakopoulos *et al.*, 2010 ▸) and the SPSB orthologue GUSTAVUS from *D. melanogaster* (PDB entry 2ihs; Woo, Imm *et al.*, 2006 ▸; Woo, Suh *et al.*, 2006 ▸). The match with highest structural similarity was the SPRY domain of Ash2L (PDB entry 3toj; Chen *et al.*, 2012 ▸), with a *Z*-score of 25.8 and an overall r.m.s. deviation of 1.6 Å on C^α^ positions for the alignment of 170 residues, although hDSPRY and Ash2L SPRY share only 23.8% sequence identity. Notably, the Ash2L SPRY domain had also been identified as a close homologue by a *BLAST* search of the PDB and its structure had been used as a search model for molecular replacement. The high *Z*-score confirms the suitability for molecular replacement in retrospect.

**Figure 4:**
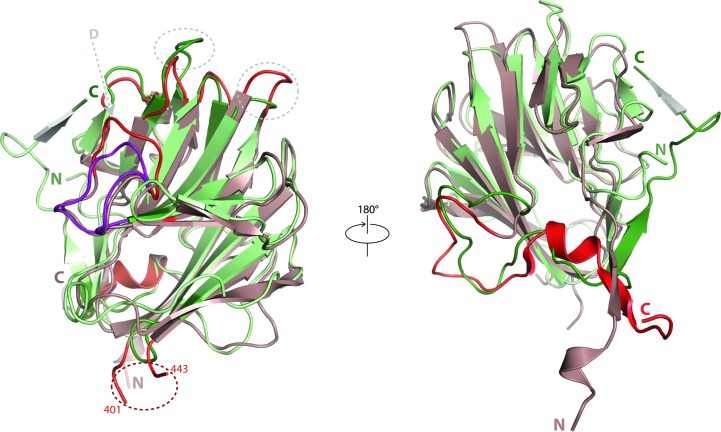
Structural comparison of hDSPRY (green) with the SPRY domain of Ash2L (red; PDB entry 3toj; Chen *et al.*, 2012 ▸) using the *DALI* server (Holm & Rosenström, 2010 ▸). Regions that show most significant structural differences are indicated by intense colour shading and are marked by grey circles. The 44-residue loop of Ash2L that is not resolved in the crystal structures is marked with a red circle. Loop D is highlighted in purple.

Whereas the core structures of hDSPRY and Ash2L SPRY are structurally very similar [besides minor differences in length in the loop regions between β3 and β4 (three residues) and between β6 and β7 (three residues) and in loop D (three residues)], hDSPRY does not harbour extensive loop insertions that are comparable in length to the loop insertions in Ash2L SPRY (Fig. 4 ▸). The longest loop observed in hDSPRY contains 23 residues and connects β-strands β14 and β15 at the C-terminus. However, a large 44-residue loop insertion connects β-strands β11 and β12 in Ash2L SPRY (Chen *et al.*, 2012 ▸). Moreover, in Bre2, a homologue of Ash2L from *Saccharomyces cerevisiae*, a 120-residue loop insertion in this region has been described (Chen *et al.*, 2012 ▸). In contrast, this loop is formed by only a four-residue loop in hDSPRY.

In hDSPRY, the C-terminus contributes to β-sheet 3 together with β-strand 15. In contrast, Ash2L SPRY lacks this β-strand as the C-terminus comes together with the N-terminus to form a small β-sheet that extends away from the β-sandwich and is not part of the SPRY domain. This ‘β-sheet extension’ adds a tail to the compact SPRY domain core and generates an overall tadpole-like structure (Chen *et al.*, 2012 ▸). In the structural model of hDSPRY the N- and C-termini do not directly interact. Nevertheless, the N- and C-terminal regions are in spatial proximity, but it remains unclear how they could form a linker that connects the inserted SPRY domain to the RecA-like core fold of DDX1, as the first 14 residues of hDSPRY are disordered in the crystal structure. The recent structures of RyR SPRY2, the second SPRY domain of RyRs, also ranked highly in the *DALI* search results, especially mouse RyR2 SPRY2 (PDB entry 4p9i; Lau & Van Petegem, 2014 ▸) with a *Z*-score of 18.9 and an overall C^α^ r.m.s. deviation of 1.9 Å for the alignment of 141 residues (27% sequence identity). The *DALI* superposition of hDSPRY with mouse RyR2 SPRY2 revealed structural differences in the N- and C-termini. Both termini are considerable longer in hDSPRY compared with mouse RyR2 SPRY2 (Supplementary Fig. S7). Interestingly, in contrast to Ash2L SPRY, in the RyR2 SPRY2 structure the termini extend away from the core domain in two opposite directions.

### 3.4. A conserved patch of positive surface charge in hDSPRY   

**Figure 5:**
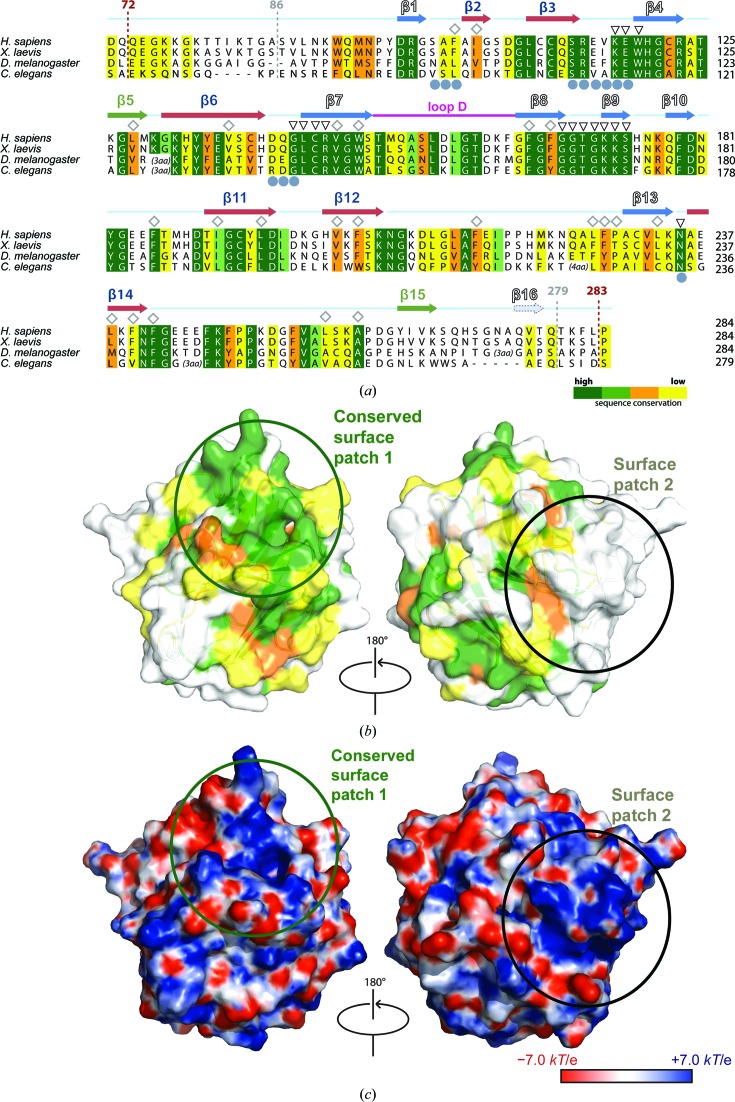
Sequence conservation of the SPRY domain amongst DDX1 homologues. (*a*) Sequence alignment of hDSPRY with the SPRY domains of DDX1 homologues from eukaryotic model organisms. Conservation values were determined using the *AMAS* server (Livingstone & Barton, 1993 ▸) and are indicated by colour coding (dark green for identical residues to yellow for less homologous residues). Residues of the hydrophobic core that stabilize the domain fold are indicated by diamonds, residues of surface A are indicated by grey circles and residues of the conserved, positively charged surface patch of hDSPRY are indicated by triangles. Secondary-structure elements are shown above the sequence alignment and are coloured according to Fig. 1 ▸. Residues that could be modelled in the crystal structure (residues 86–279) are indicated in grey and domain boundaries of the crystallization construct are indicated in brown (residues 72–283). (*b*) Sequence conservation mapped onto the molecular surface of hDSPRY. (*c*) Electrostatic surface potential, calculated using *APBS* (Baker *et al.*, 2001 ▸), mapped onto the molecular surface.

DDX1 is widespread in eukaryotic organisms and, in addition to the RecA-like domains (Supplementary Fig. S4), the SPRY domain is also highly conserved (Fig. 5 ▸ *a*). The residues of the hydrophobic core stabilizing the β-sandwich fold in hDSPRY were found to be either conserved or substituted with similar hydrophobic residues (Fig. 5 ▸ *a*). The residues of most β-strands are conserved, except for those of two β-strands at the C-terminus: β-strand β15 of β-sheet 3 and the potentially artificial strand β16. Interestingly, the degree of conservation varies between the two sheets, and residues that are part of β-sheet 1 are virtually identical in DDX1 orthologues, whereas the residues of β-sheet 2, mostly of strands β2, β12 and β14, are less conserved (Fig. 5 ▸ *a*). Nevertheless, the residues of the hydrophobic core are still conserved. Notably, the N- and C-terminal regions of the SPRY domain are not conserved at all and belong to the few loop regions that significantly differ in amino-acid sequence and length (4–9 residues) in DDX1 (Supplementary Fig. S4). The residues at these termini correspond to the regions that connect the compact SPRY domain to the RecA-like domain 1 of the DDX1 protein core. Conservation of these linker regions is most likely to be dispensable for DDX1 function, and thus these regions lack any evolutionary selective pressure for sequence maintenance. In addition to these linker regions, most of the loop regions in hDSPRY are also not well conserved and differences in the number of residues are also found in some of the loop regions (Fig. 5 ▸ *a*).

When the sequence conservation within DDX1 from different species was mapped onto the molecular surface of the hDSPRY structure, a highly conserved surface patch was identified (surface patch 1 in Fig. 5 ▸ *b*). This patch is mainly formed by conserved residues of β-sheet 1, the adjacent loop regions and the N-terminal part of loop D (Fig. 5 ▸ *b*). The accessibility of the surface patch raises the question whether this conserved patch might be a protein–protein interaction platform in hDSPRY. In fact, the binding sites of SPRY domains are highly conserved across species and homologous proteins are functionally interchangeable (Keeble *et al.*, 2008 ▸; Filippakopoulos *et al.*, 2010 ▸; D’Cruz, Kershaw *et al.*, 2013 ▸). Further support for this hypothesis comes from the electrostatic surface potential of hDSPRY, which revealed that this conserved region is positively charged (surface patch 1 in Fig. 5 ▸ *c*). As proposed for other SPRY domains (Filippakopoulos *et al.*, 2010 ▸; James *et al.*, 2007 ▸; Kuang *et al.*, 2009 ▸), hDSPRY might establish protein–protein interactions through electrostatic interactions. A second, positively charged patch is located at the N-terminus of hDSPRY (surface patch 2 in Fig. 5 ▸ *c*) that is part of the linker regions; however, this region is not conserved in DDX1.

### 3.5. Comparison to interaction surfaces in other SPRY domains   

Most of the SPRY domains have been proposed to serve as protein–protein interaction platforms (D’Cruz, Babon *et al.*, 2013 ▸) and it is thus conceivable that hDSPRY serves a similar role by using its conserved surface patch. The first structural study that described the interaction of a SPRY domain with its binding partner in atomic detail was the SPRY domain of GUSTAVUS (PDB entry 2ihs; Woo, Suh *et al.*, 2006 ▸). In this crystal structure, a 20-residue peptide derived from the DEAD-box helicase VASA was bound to loop regions connecting the two β-sheets. Since then, similar structures, such as those of the SPRY domains of the homologous mammalian SPSB proteins 1, 2 and 4 (Filippakopoulos *et al.*, 2010 ▸; Kuang *et al.*, 2009 ▸), have been reported. GUSTAVUS and the homologous SPSB SPRY domains revealed a common mode of interaction between these SPRY domains and their cognate peptides, and a common surface patch, termed surface A, has been established as an inter­action platform (Woo, Suh *et al.*, 2006 ▸; Filippakopoulos *et al.*, 2010 ▸). Surface A is formed by the loops that connect β-sheets 1 and 2 on one site of the β-sandwich and this surface patch is extended by loop D, which connects the β-sandwich at the other side.

**Figure 6:**
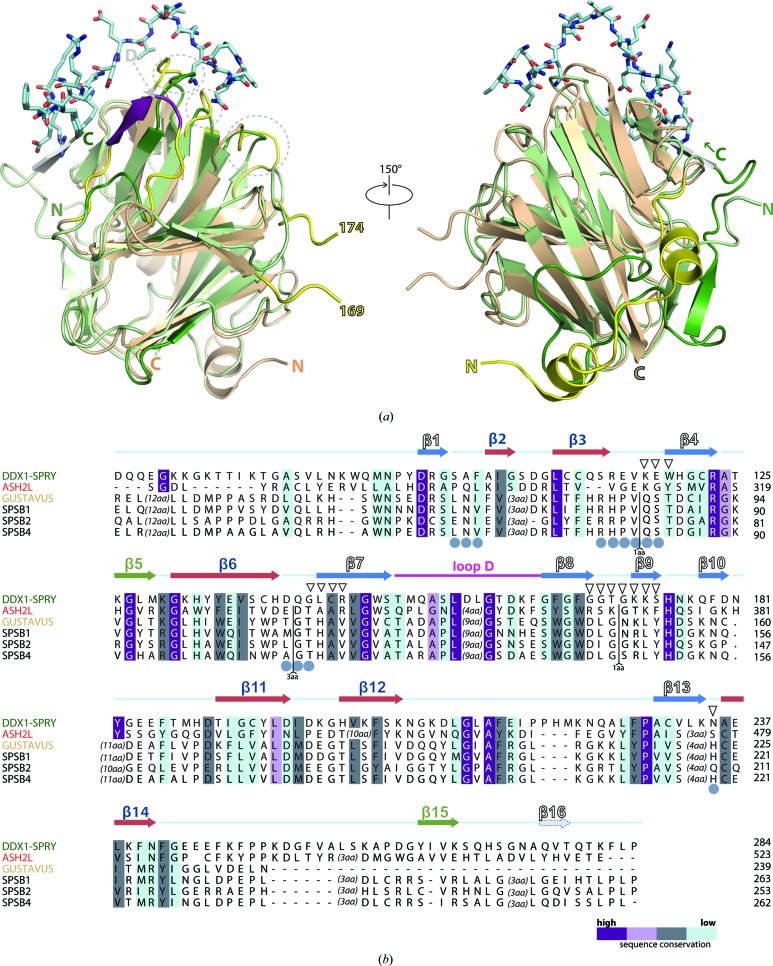
Comparison of the interaction surface of SPRY complex structures. (*a*) *DALI* superposition of hDSPRY (green) with the SPRY domain of *D. melanogaster* GUSTAVUS (yellow) and a 20-residue VASA peptide (shown as a stick model; PDB entry 2ihs; Woo, Suh *et al.*, 2006 ▸). Loop D is marked in purple. The loop region between residues 169 and 174 in GUSTAVUS is not resolved. (*b*) Sequence alignment of hDSPRY with the best hits from the *DALI* search. Conservation values were determined using the *AMAS* server (Livingstone & Barton, 1993 ▸) and are indicated by colour coding (from dark purple for identical residues to light blue for less homologous residues). Residues of surface A of related SPRY domains are labelled with grey circles, and residues of the conserved, positively charged surface patch of hDSPRY are labelled with triangles.

Notably, the SPSB domain of GUSTAVUS, the prototype of a surface A-containing SPRY domain, was found to be structurally closely related to hDSPRY during the *DALI* search (*Z*-score of 18.3 and 2.0 Å r.m.s. deviation of C^α^ atoms on the alignment of 152 residues with 24% amino-acid sequence identity). Structural differences between hDSPRY and GUSTAVUS SPRY (with a peptide bound) after superposition by *DALI* were found in the loops that form surface A in GUSTAVUS SPRY, with differences in the backbone trace of between 3.2 and 5.4 Å (Fig. 6 ▸ *a*). However, the observed differences are not caused by the bound peptide in the GUSTAVUS SPRY structure, as no significant changes were observed between the peptide-bound and free GUSTAVUS SPRY structures [PDB entries 2ihs (Woo, Suh *et al.*, 2006 ▸) and 2fnj (Woo, Imm *et al.*, 2006 ▸), respectively]. Although a flexible nature of loop D could be suggested by the recent structure of the SPRY2 domain of mouse RYR2 (PDB entry 4p9i; Lau & Van Petegem, 2014 ▸), in which loop D was not resolved, comparison of the apo and peptide-bound forms of GUSTAVUS SPRY shows that peptide binding does not fix loop D of surface A. Interestingly, loop D is much shorter in hDSPRY than in GUSTAVUS SPRY and cannot form part of the putative surface A in hDSPRY. Furthermore, the residues that form surface A are conserved in all SPSB SPRY domains but differ in hDSPRY and Ash2L SPRY (Fig. 6 ▸ *b*). Moreover, the sequence alignment of different DDX1 SPRY domains showed that those loop regions are not conserved among different DDX1 homologues. Although the conserved surface patch found for hDSPRY has a minor spatial overlap with surface A, it seems that DDX1 might bind its partner differently from the mode established from SPSB complex structures. Loop regions that form surface A in GUSTAVUS and related SPSB proteins were also shown to form the protein–protein interaction surface in the complex structures of SPRY domains of the more distantly related TRIM21 proteins (James *et al.*, 2007 ▸; Keeble *et al.*, 2008 ▸). Although the interaction surface is established by equivalent loops in TRIM and SPSB proteins, the overall architecture differs owing to length and conformational differences in these loops. Nevertheless, these previously characterized interaction loops show no significant overlap with the conserved surface patch in the SPRY domains of DDX1.

In conclusion, our work presents the first structural information on the human DDX1 DEAD-box protein, which is an essential player in cellular RNA processing. We have determined a high-resolution structure of hDSPRY, the domain that is not part of the canonical DEAD-box protein core. The structure shows two layers of concave-shaped β-sheets that stack to together to form a compact β-sandwich conformation covered by a third small β-sheet. We have discovered a positively charged surface region in hDSPRY that is highly conserved in DDX1 across species. This surface patch might constitute the protein–protein interaction site within hDSPRY. This potential interaction site differs from the loops that mediate protein–protein interactions in complex structures of other SPRY domains. Notably, evidence is accumulating that the interaction of DEAD-box proteins with their cognate binding partners is frequently mediated by the SPRY domains of the latter (Kowalinski *et al.*, 2011 ▸; Zhang *et al.*, 2013 ▸; Woo, Imm *et al.*, 2006 ▸). In contrast to other DEAD-box proteins, DDX1 has a SPRY domain integrated in its polypeptide chain and it is conceivable that SPRY-domain insertion will directly regulate its correct assembly into DDX1-dependent multiprotein complexes (Popow *et al.*, 2011 ▸; Han *et al.*, 2014 ▸). DDX1 is an important factor in the replication of HIV-1, and its SPRY domain is likely to be an important structural motif that mediates the specific interaction of DDX1 with other factors such as the HIV-1 Rev protein. Notably, the N-terminal region of DDX1 exclusively contains the Rev-binding domain (Edgcomb *et al.*, 2011 ▸), and it is conceivable that hDSPRY mediates this interaction. Therefore, our structure provides a basis for the detailed biochemical characterization of the hDSPRY interaction platform and the mechanisms of the recruitment of DDX1 to hetero-oligomeric complexes.

## 4. Related literature   

The following reference is cited in the Supporting Information for this article: Story *et al.* (2001 ▸). 

## Supplementary Material

PDB reference: SPRY domain of human DDX1, 4xw3

Supporting Information.. DOI: 10.1107/S2053230X15013709/cb5085sup1.pdf

## Figures and Tables

**Table 1:** Statistics of data collection and refinement (molecular replacement) Values in parentheses are for the highest resolution shell.

PDB code	4xw3
X-ray source	Beamline X10SA, PSI
Wavelength ()	1.070
Space group	*P*2_1_2_1_2_1_
Unit-cell parameters (, )	*a* = 45.06, *b* = 76.14, *c* = 122.66, = = = 90
Resolution range ()	502.0 (2.12.0)
Observed reflections	367329 (48403)
Unique reflections	28891 (3815)
Multiplicity	12.7 (12.7)
*I*/(*I*)	23.6 (6.5)
Completeness (%)	98.6 (97.6)
*R*_meas_ † (%)	7.9 (58.6)
Wilson *B* factor (^2^)	32.7
Refinement statistics	
Resolution ()	47.782.0
No. of reflections used in refinement	27446
No. of reflections used for calculation of *R*_free_	1445
*R*_work_/*R*_free_ ‡ (%)	20.0/23.7
No. of non-H atoms	
Total	3144
Protein	3002
Water molecules	142
Average *B* factors (^2^)	
Overall	28.3
Protein (chain *A*/*B*)	28.0/28.0
Water molecules	34.0
R.m.s. deviations from ideal geometry	
Bond lengths ()	0.009
Bond angles ()	1.139
Ramachandran plot	
Most favoured regions (%)	88.1
Additional allowed regions (%)	11.9
Generously allowed regions (%)	0.0
Disallowed regions (%)	0.0

† *R*_meas_ = 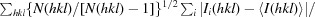 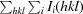, where *I*(*hkl*) is the mean intensity of symmetry-equivalent reflections and *N*(*hkl*) is the redundancy.

‡ *R*_work_ = 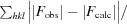 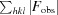 (working set, no cutoff applied); *R*_free_ is the *R* value calculated for 5% of the data set that was not included in refinement.
