# Supplementary material for: Structure of the SPRY domain of the human RNA helicase DDX1, a putative interaction platform within a DEAD-box protein
Source: Acta Crystallogr F Struct Biol Commun. 2015 Aug 25;71(Pt 9):1176–88. doi: 10.1107/S2053230X15013709 (PMC4555926; doi:10.1107/S2053230X15013709)
Supplement: Supplementary file 1 [file f-71-01176-sup1.pdf]

STRUCTURAL BIOLOGY
COMMUNICATIONS

Volume 71 (2015)

Supporting information for article:

Structure of the SPRY domain of human DDX1 helicase, a putative interaction platform within a DEAD-box protein

Julian Kellner and Anton Meinhart

Table S1 Different protein constructs used for structure determination.

Construct length is given with residue numbers referring to DDX1.

construct	length	solubility/ crystals	space group	unit cell parameters (Å,°)	Resolution (Å)
SPRY_72-283+Tag	72-283	+/+	$P2_12_12_1$	*a* = 43.73, *b* = 75.64, *c* = 122.73, α = β = γ = 90	4.0
SPRY_72-283	72-283	+/+	$P2_12_12_1$	*a* = 45.06, *b* = 76.14, *c* = 122.66, α = β = γ = 90	2.0
SPRY_84-283+Tag	84-283	+/-			
SPRY_100-283+Tag	100-283	-			
SPRY_72-261+Tag	72-261	+/-			
SPRY_84-261+Tag	84-261	+/+	*P*1	*a* = 35.47, *b* = 99.28, *c* = 136.36, α = 102.56, β = 97.62 γ = 98.33	2.7
SPRY_84-261+Tag (SeMet)	84-261	+/+	$P2_1$	*a* = 37.14, *b* = 133.80, *c* = 35.96, α = γ = 90, β = 118.26	3.4
SPRY_100-261+Tag	100-261	-			

Table S2 Primers used for cloning of the DDX1 SPRY domain and for site-directed-mutagenesis to truncate the N- and C-terminus (mutations are underlined, introduced nucleotides are italic)

name	Sequence [5’ -> 3’]	application
**DDX1_NheI_fwd**	GGGAAGGGCTAGCATGGCGGCCTTCTCCG<br>-> region of DDX1	Amplify the DDX1 ORF, primer contains NheI site for cloning into pET28a
**DDX1_NotI_rev**	GGGATCGCGGCCGCTCAGAAGGTTCTGAACAGCTG<br>-> region of DDX1	Amplify the DDX1 ORF, primer contains NotI site for cloning into pET28a
**SPRY_ BamH_fwd**	GTTTATGAAACTCTGAAAGG*ATCC*CAGGAAGGCAAAAAAG	Generate BamHI site after Lys69 to clone SPRY-coding region of DDX1
**SPRY_ BamH_rev**	CTTTTTTGCCTTCCTG*GGAT*CCTTTCAGAGTTTCATAAAC	see above
**SPRY_ XhoI_fwd**	CAAACAAAGTTTCTC*GA*GAATGCTCCGAAAGCTC	Generate XhoI site downstream of the SPRY domain to clone it as BamHI-XhoI fragment
**SPRY_ XhoI_rev**	GAGCTTTCGGAGCATTC*AG*GAGAAACTTTGTTTG	see above
**SPRY_Gly84_BamHI_fwd**	GAAAAACAACAATTAAAG*GATCC*GCTTCAGTGCTG	Generate BamHI site after Gly84 to truncate N-terminus of SPRY domain
**SPRY_Gly84_BamHI_rev**	CAGCACTGAAGC*GGATC*CTTTAATTGTTGTTTTTC	see above
**SPRY_Ser100_BamHI_fwd**	CATATGACAGA*GGATC*CGCTTTTGCAATTGGG	Generate BamHI site after Ser100 to truncate N-term of SPRY domain
**SPRY_Ser100_BamHI-rev**	CCCAATTGCAAAAGCG*GATCC*TCTGTCATATG	see above
**SPRY_Ala261_Stop_fwd**	CTCTTTCCAAGGCAT*GATAA*GGTTACATTG	Generate double stop-codon after Ala261 to truncate C-term of SPRY domain
**SPRY_Ala261_Stop_rev**	CAATGTAACC*TTATC*ATGCCTTGGAAAGAG	see above

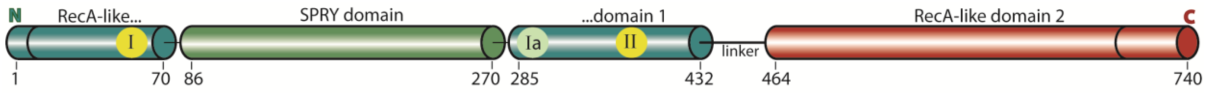

Figure S1 Domain organization of human DDX1 is shown schematically. Position of conserved helicase motifs I, Ia and II is indicated.

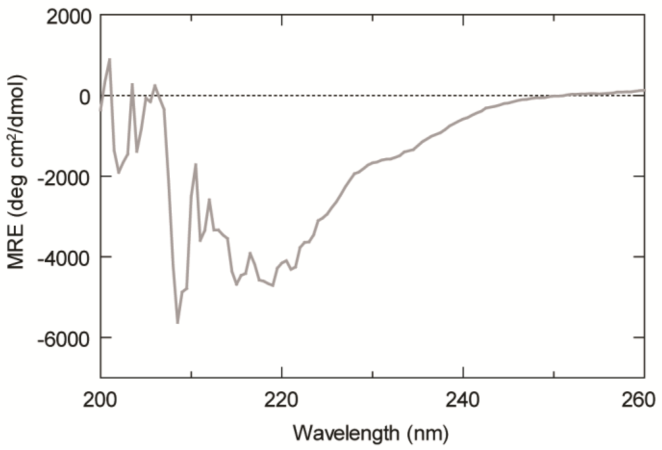

Figure S2 CD spectrum of construct SPRY_72-283 at 130 µg/ml (5 µM) protein concentration in CD buffer (50 mM K_2HPO_4/KH_2PO_4 pH 8.0, 250 mM KF, 3 mM DTE) is shown. Please note that UV signals is noisy in the far-UV (below 215 nm) regime. Data traces represent the average of five spectra and the CD signal was converted to mean-residue-ellipticity (MRE).

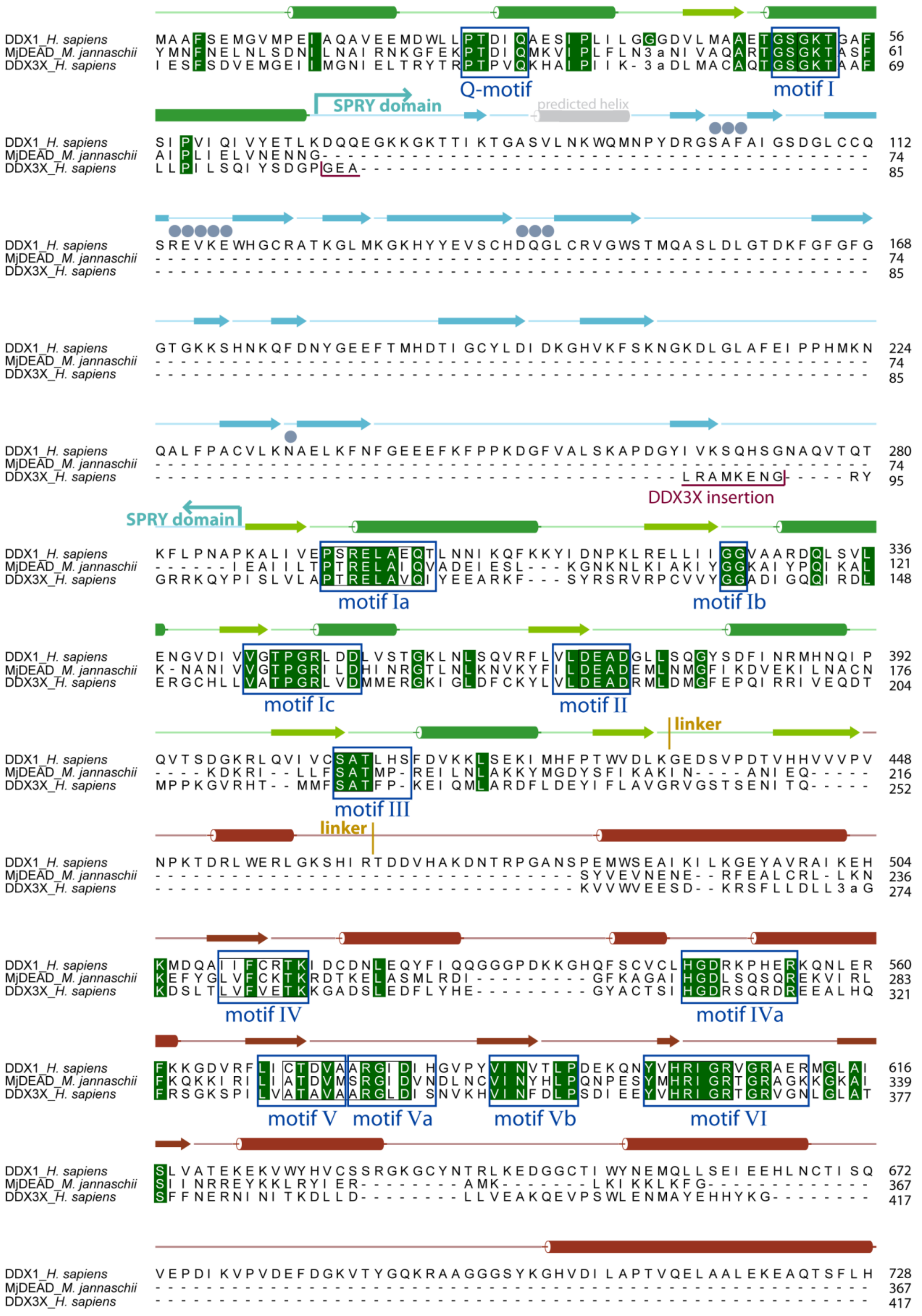

Figure S3 Amino acid sequence alignment of DDX1 and homologous DEAD-box proteins MjDEAD from *Methanococcus jannaschii* (Story *et al.*, 2001) and DDX3X from *Homo sapiens*

(Hogbom *et al.*, 2007). Identical residues are highlighted in green. Secondary structure elements (arrows indicating β-strands, tons indicating α-helices) as predicted for human DDX1 by PSIPRED (Buchan *et al.*, 2010) are shown above the sequence. Colouring is performed according to domain allocation; RecA-like domain 1 is depicted in green; the SPRY domain is depicted in blue; the RecA-like domain 2 is depicted in red. A DDX3X specific insertion, which forms an elongated positively charged cavity(Hogbom *et al.*, 2007), is indicated in dark red.

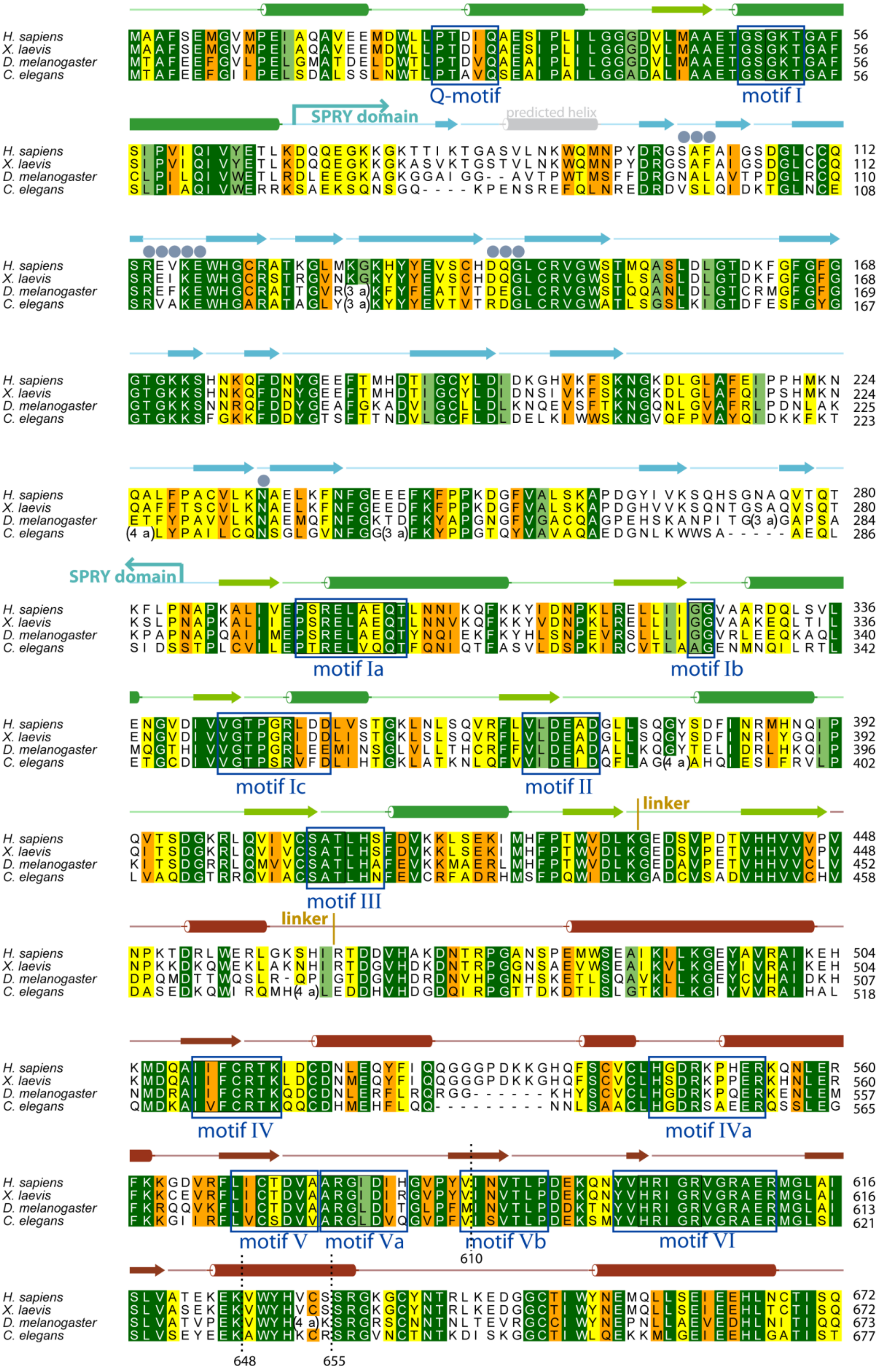
H. sapiens
X. laevis
D. melanogaster
C. elegans
Q-motif
motif I
SPRY domain
predicted helix
motif Ia
motif Ib
motif Ic
motif II
linker
motif III
motif IV
motif IVa
motif V
motif Va
motif Vb
motif VI
610
648
655

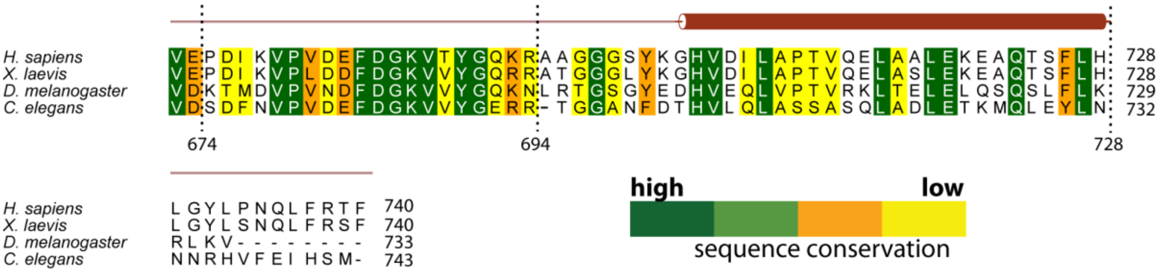

Figure S4 Amino acid sequence alignment of DDX1 orthologs from representative eukaryotic model organisms. Conservation values were determined using the AMAS server (Livingstone & Barton, 1993) and are indicated by color coding (dark green for identical residues to yellow for less homologous residues). Secondary structure elements (arrows indicating β-strands, tubes indicating α-helices) as predicted for human DDX1 by PSIPRED (Buchan *et al.*, 2010) are shown above the sequence. Colouring is performed according to domain allocation; RecA-like domain 1 is depicted in green; the SPRY domain is depicted in blue; the RecA-like domain 2 is depicted in red.

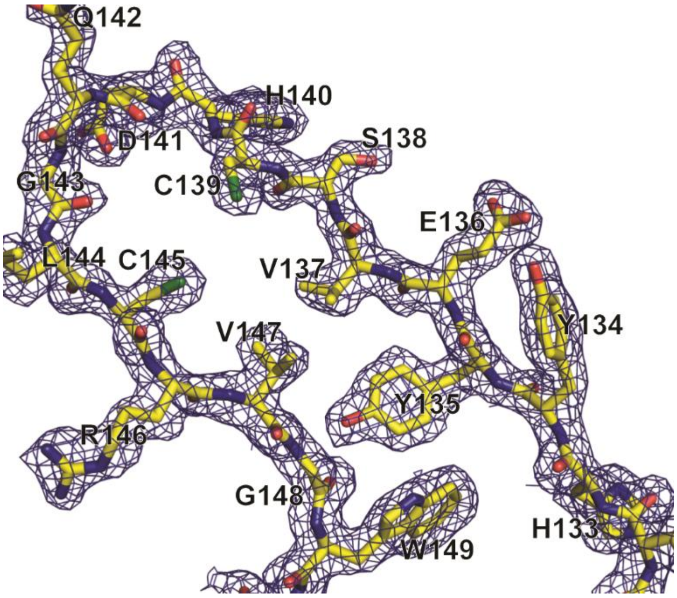

Figure S5 A fully-refined $2F_o$-F_c electron density map contoured at 1σ sigma covering the strands β6 and β7.

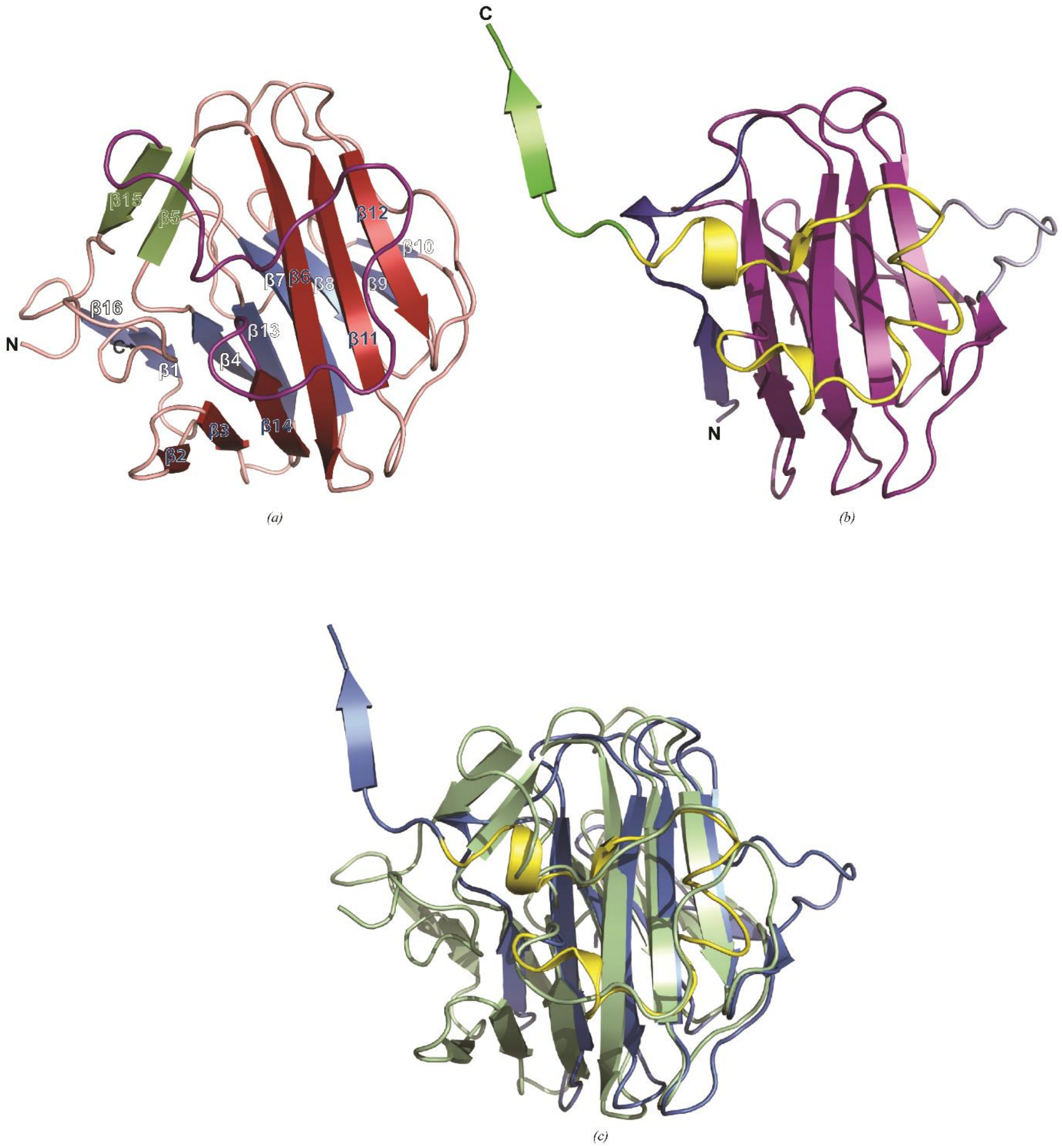

Figure S6 A potential lid loop similar as described for murine RyR2 SPRY2 (Lau & Van Petegem, 2014) that covers the hydrophobic surface at a convex side of β-sheet 2 of the SPRY domain of DDX1. (*a*) DDX1 colored similar as in **Fig. 2** but rotated horizontally by 180°. The lid loop is highlighted in purple. (*b*) Structure of murine RyR2 SPRY2 (PDB entry 4p9i). The N-terminal extension in depicted in blue, the SPRY domain core in purple, the insertion loop in light blue, the lid in yellow; and the C-terminal tail in green; the domain assignment is adopted from (Lau & Van Petegem, 2014). (*c*) A DALI superposition (Holm & Rosenstrom, 2010) of hDSPRY (green) with the structure of murine RyR2 SPRY2 (blue). The lid loop of murine RyR2 SPRY2 is highlighted in yellow.

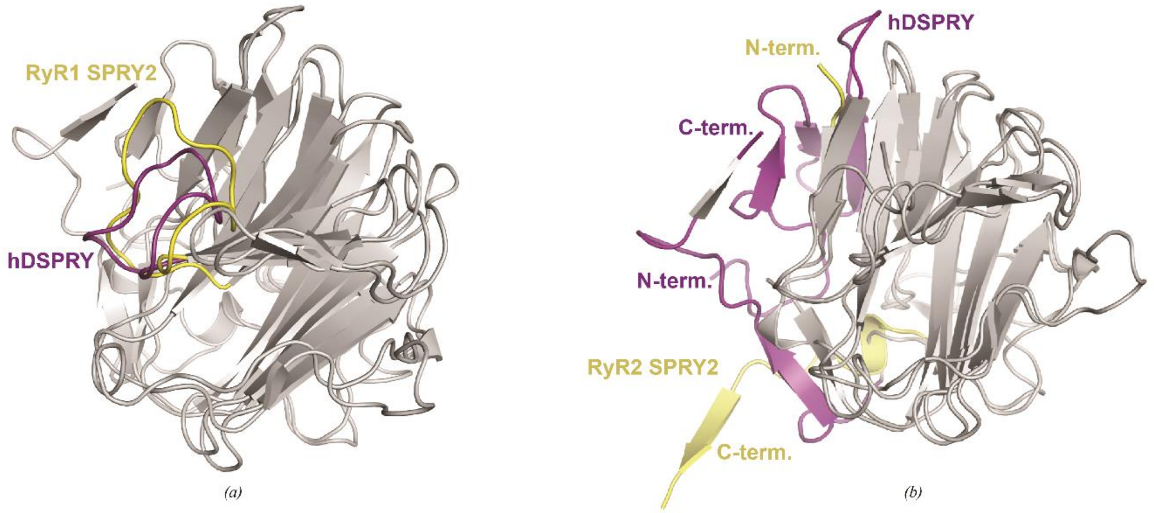

Figure S7 A DALI superposition (Holm & Rosenstrom, 2010) of hDSPRY with rabbit and mouse RyR SPRY2 domains (Lau & Van Petegem, 2014). (*a*) Superposition of hDSPRY with rabbit RyR1 SPRY2 (PDB entry 4p9j); Loop D is highlighted in purple in case of hDSPRY or RyR1 SPRY2 in yellow, respectively. The common SPRY core of both domains is shown in gray. (*b*) Superposition of hDSPRY with mouse RyR2 SPRY2 (PDB entry 4p9i). Differences in the length and orientation of the N- and C-terminal regions are highlighted purple in case of hDSPRY or RyR2 SPRY2 in yellow, respectively. The common SPRY core of both domains is shown in gray.

Buchan, D. W., Ward, S. M., Lobley, A. E., Nugent, T. C., Bryson, K. & Jones, D. T. (2010). *Nucleic Acids Res* **38**, W563-568.
Hogbom, M., Collins, R., van den Berg, S., Jenvert, R. M., Karlberg, T., Kotenyova, T., Flores, A., Karlsson Hedestam, G. B. & Schiavone, L. H. (2007). *J Mol Biol* **372**, 150-159.
Holm, L. & Rosenstrom, P. (2010). *Nucleic Acids Res* **38**, W545-549.
Lau, K. & Van Petegem, F. (2014). *Nat Commun* **5**, 5397.
Livingstone, C. D. & Barton, G. J. (1993). *Comput Appl Biosci* **9**, 745-756.
Story, R. M., Li, H. & Abelson, J. N. (2001). *Proc Natl Acad Sci U S A* **98**, 1465-1470.
